# Analysis of latent tuberculosis and mycobacterium avium infection data using mixture models

**DOI:** 10.1186/1471-2458-6-240

**Published:** 2006-09-28

**Authors:** José I Villate, Berta Ibáñez, Valentín Cabriada, José I Pijoán, Jorge Taboada, Arantza Urkaregi

**Affiliations:** 1 Preventive Medicine Department, Cruces Hospital, Barakaldo, Spain; 2 Preventive Medicine and Public Health Department, Basque Country University, Leioa, Spain; 3 Basque Foundation for Health Innovation and Research, Sondika, Spain; 4 Neumology Department, Cruces Hospital, Barakaldo, Spain; 5 Clinical Epidemiology Unit, Cruces Hospital, Barakaldo, Spain; 6 Mathematics Department, Science Faculty, Basque Country University, Leioa, Spain

## Abstract

**Background:**

Estimation of the frequency of latent tuberculosis infection (LTBI) is difficult in areas with low tuberculosis infection rates and high exposure to non-tuberculous mycobacteria (NTM), including BCG vaccination. The objective was to assess LTBI and M avium infection and to estimate their probability based on skin tests responses in an infant population from a region with the aforementioned characteristics.

**Methods:**

A population-based tuberculin skin test (TST) and sensitin (M avium) survey was conducted on seven years old infants in Biscay, a province from The Basque Country (Spain). 2268 schoolchildren received sensitin and 5277 TST. Participation rate was 89%. Commonly used estimation methods were compared with a method based on the fit of mixture models using the Expectation Maximization algorithm. Functions estimating the probabilities of LTBI and M avium infection given the observed skin tests responses were developed for vaccinated and unvaccinated children.

**Results:**

LTBI prevalences varied widely according to the estimation method. The mixture model provided prevalences higher than expected although intermediates between those obtained by currently recommended approaches. Exposure to previous BCG vaccine produces an upward shift of an average of about 3 mm on the induration size to attain the same probability of infection.

**Conclusion:**

Our results confirm the commonplace exposure to NTM which effect should be taken into account when performing and assessing tuberculin surveys. The use of mixture analysis under the empirical Bayes framework allows to better estimate the probability of LTBI in settings with presence of other NTM and high BCG-vaccination coverage. An estimation of the average effect of BCG vaccination on TST induration is also provided. These models maximise information coming from classical tuberculin surveys and could be used together with the newly developed blood tests to improve survey's specificity and cost-effectiveness.

## Background

Tuberculosis is a well-recognised worldwide health problem [1]. However, the picture differs dramatically depending on the socioeconomic development of the area in question. While in developing countries tuberculosis is still a first order challenge, it is being kept increasingly under control in developed countries. In the latter, the diagnosis and treatment of Latent Tuberculosis Infection (LTBI) is now necessary in order to eliminate tuberculosis [2]. New blood tests [3,4] are already changing the approach to this problem, and are to be implemented in the near future in western industrialised countries. However, tuberculin skin tests (TST) remain today the most used tools for LTBI diagnostic [5] and treatment [2] decisions at a global scale.

Unfortunately, the interpretation of TST results suffers from several drawbacks when performed on populations where the BCG vaccination rate is high [6]. This situation becomes even more awkward when cross-reactivity due to infection with nontuberculous mycobacteria (NTM) is present [7,8] (a subject which has not been addressed much so far) and the survey is carried out in a region with low tuberculosis prevalence [9].

In the region under study, a province in the Basque Country (Spain), all the aforementioned characteristics that make the interpretation of TST results difficult are present. The Basque Country is the only community that has had a programme of systematic BCG vaccination in use for more than two decades [10], in spite of being one of the communities with the highest socioeconomic level. Despite the current debate regarding whether it is appropriate to maintain this policy in our region due to a substantial decline in tuberculosis prevalence rates, methods that allow a continuous and accurate monitoring of LTBI in settings of this kind are still required.

Several statistical approaches have been attempted to cope with the confounding effect of NTM infection (BCG vaccination included) in the accurate estimation of LTBI. Traditionally, methods that propose fixed general cut-off points have been in use [11-14]. However, problems in determining the prevalence of LTBI using these methods have long been recognised, making the exploration of new statistical methods [7] necessary. Along these lines, Neuenschwander et al. [15-17] have applied the so-called mixture model analysis [18] to this problem, first focusing on unvaccinated individuals and then extending this approach to BCG-vaccinated subjects. They have shown mixture models to be a flexible tool for analysing data of this kind.

The purpose of the present study is threefold: a) to estimate the prevalence of LTBI and *Mycobacterium avium *(*M avium*) infection in the infant population of The Basque Country (Spain), b) to find a method that could help us to accurately discriminate LTBI from infection with other mycobacteria, including BCG vaccination and c) to help determine the probability of LTBI and *M avium *infection according to tuberculin or sensitin induration sizes respectively.

## Methods

### Population and sample size

The region under study is called Biscay, a province of The Basque Country with a population of 1.2 million inhabitants. In 1997, 13 smear-positive sputum cases among the total 38 incident tuberculosis cases per 100,000 population were notified in the region [19]. Our study population consisted of all children living in Biscay and turning seven years of age during 1998. We decided to study school-age children based on two grounds: to allow for an effect of the BCG vaccination to still be present and accessibility criteria. This accounts for 8430 children.

To estimate the required sample size, we used results from a previous survey in the same region [20]. A vaccination coverage rate of 89%, a participation rate of 80%, a maximum prevalence of 20% among BCG-vaccinated and 2% among unvaccinated children, respectively, with TST induration size ≥ 10 mm were anticipated. Standard errors of 0.4 and 0.35% were respectively accepted for the prevalence estimations. A required sample size of 6250 children was obtained, a quantity close to the total population size assuming a recruitment rate of 80%, so eventually we decided to recruit as many schoolchildren as possible, since the study could provide the potential benefit of detecting additional children with very large indurations, requiring further examination in order to assess the need for prophylactic treatment.

### Survey design

This study focuses on some aspects of a more general research project devoted to investigate the distribution of tuberculin and sensitin induration sizes as well as the relationship among them and the so-called booster effect. In order to accomplish this endeavour, recruited children were randomly distributed in three groups. Two thirds received tuberculin as the first test and one third received the *M avium *sensitin, which was the most prevalent MNT in our region at the study time (data provided by the Basque Country Public Health Department). Out of the children receiving tuberculin as the first test, half were given a repeat dose of tuberculin one week later, and half were given the sensitin. Children who had received the sensitin as the first test were given a tuberculin one week later. In this paper, only results regarding administration of the first skin test are shown.

The field work was planned to be conducted in 5 months so as to prevent substantial changes in the children's characteristics and personnel involved in the process that could influence results.

Firstly, an informative campaign among parents and teachers was conducted, presenting the aim and methods of the study, and encouraging them to participate. In addition, an informative leaflet was handed out. Parents had to give written informed consent prior to inclusion of their children in the study. The vaccine information recorded in the Personal Paediatric Record was requested. The protocol was approved by the hospital's Research Ethics Committee.

There were two criteria that led to children not receiving the skin test: absence of written informed permission and the existence of documented evidence of previous positive TST.

Demographic data, past medical history and vaccination status were recorded at study entry. As the latter was deemed a key point in this research, we used three methods to improve its classification: a) certificate of BGC vaccination in the Personal Paediatric Record; b) confirmation of BGC vaccination in the province register; c) BGC scar. To classify a child in the non-BCG group, all three methods had to have negative results and to classify a child as BCG-vaccinated, at least two of three criteria had to be positive. Those who did not fulfil these requirements were considered to have an unknown vaccination status.

### Skin tests

Intradermal injections with 0.1 ml of 2 TU PPD-RT23 tuberculin (bioequivalent to 5 TU PPD-S) and 0.1 ml of RS-10 (M. avium) sensitin were used. The PPD RS-10 was chosen because it is the most frequently isolated NTM among the patients in our area. All tuberculin doses belonged to the same lot (L-03) and sensitin doses came from two lots (L-66 and L-68) [21]. Both tuberculin and sensitin were prepared in and obtained from Statens Serum Institut in Copenhagen, Denmark.

The tuberculin skin test was administered intradermally [22] and the induration diameter was measured transversely to the long axis of the arm 72 hours after application.

Test application and reading were performed by a specifically trained team led by a specialist in respiratory medicine and carried out by eight highly experienced nurses. Four two-nurse groups were assembled, and each group performed double and independent readings.

### Statistical analysis

#### Descriptive analysis

The distribution of induration diameters was described graphically using histograms and categorised using 5 mm-intervals. The prevalence of LTBI was estimated using three methods proposed in the literature [14,23,24]. The first criterion, probably the most widely used, considers all reactions ≥ 10 mm to be a marker of LTBI [14]. The second one multiplies the proportion of reactions of size ≥ 14 mm by 1.22 [24], and the last one, also known as the mirror method, considers all indurations of 17 mm counted once and indurations of ≥ 18 mm counted twice to obtain the estimated number of infections [23,24].

#### Prevalence estimation using mixture models

This statistical approach has been extensively used in situations where a population is composed of several subgroups and the main issue is to find out which subgroup one individual belongs to [25]. Neuenschwander et al [15] applied this method to the analysis of LTBI prevalence, initially restricted to an unvaccinated population and later on was extended to BCG-vaccinated populations [16,17].

In our setting there are four possible subgroups according to tuberculin induration response: individuals with null induration after intradermal injection, individuals with reaction due to NTM, individuals with reaction due to previous BCG vaccination and individuals with reaction due to LTBI. Therefore, for BCG-vaccinated children the mixture model will be comprised of four components in the following sequential order [16]:

*y*_*j *_≈ *p*_0 _I{yj=0}
 MathType@MTEF@5@5@+=feaafiart1ev1aaatCvAUfKttLearuWrP9MDH5MBPbIqV92AaeXatLxBI9gBaebbnrfifHhDYfgasaacH8akY=wiFfYdH8Gipec8Eeeu0xXdbba9frFj0=OqFfea0dXdd9vqai=hGuQ8kuc9pgc9s8qqaq=dirpe0xb9q8qiLsFr0=vr0=vr0dc8meaabaqaciaacaGaaeqabaqabeGadaaakeaacqWGjbqsdaWgaaWcbaGaei4EaSNaemyEaK3aaSbaaWqaaiabdQgaQbqabaWccqGH9aqpcqaIWaamcqGG9bqFaeqaaaaa@35F7@ + *p*_1_*f*_*EM *_(*y*_*j*_, θ_1_) + *p*_2_*f*_*BCG *_(*y*_*j*_, θ_2_) + *p*_3_*f*_*TB *_(*y*_*j*_, θ_3_)     (1).

where *y*_*j *_represents the induration (in millimetres) for the *j*^*th *^individual, I{yj=0}
 MathType@MTEF@5@5@+=feaafiart1ev1aaatCvAUfKttLearuWrP9MDH5MBPbIqV92AaeXatLxBI9gBaebbnrfifHhDYfgasaacH8akY=wiFfYdH8Gipec8Eeeu0xXdbba9frFj0=OqFfea0dXdd9vqai=hGuQ8kuc9pgc9s8qqaq=dirpe0xb9q8qiLsFr0=vr0=vr0dc8meaabaqaciaacaGaaeqabaqabeGadaaakeaacqWGjbqsdaWgaaWcbaGaei4EaSNaemyEaK3aaSbaaWqaaiabdQgaQbqabaWccqGH9aqpcqaIWaamcqGG9bqFaeqaaaaa@35F7@ is an indicator function (takes value 1 if *y*_*j *_= 0 and 0 otherwise), *f*_*EM*_, *f*_*BCG *_and *f*_*TB *_represent the distribution for each subgroup respectively: environmental mycobacteria reaction, BCG vaccination reaction and LTBI reaction, being, *p*_1_, *p*_2 _and *p*_3 _the corresponding probabilities and *p*_0 _= 1-(*p*_1_+ *p*_2_+*p*_3_) the probability associated with null induration. For unvaccinated children, the *f*_*BCG *_component is excluded resulting in a three-component mixture model. In this model, the order of the components reflects the assumption of increasing component influences on the induration sizes. A similar model was derived for the sensitin analysis, changing the component order (*f*_*BCG*_, *f*_*TB *_and *f*_*EM*_).

To analyse the tuberculin and sensitin responses, we tested different parametric distributions for each component, namely, normal, lognormal and Weibull, as suggested by Neuenschwander et al. [16]. Their Bayesian approach to parameter estimation led to convergence problems in our data, and therefore we used an alternative procedure within the empirical-Bayes framework to tackle this problem: the EM estimation algorithm [26] in the presence of incomplete data. This method need not incorporate prior information from subject-matter knowledge. Parameter confidence intervals have been derived using parametric bootstrap techniques [27], which have already been used in mixture models applications [28]. These methods were programmed in the freely available R software [29] [see Additional file 2].

## Results

### I-Participation and BCG coverage

The survey fieldwork lasted five months as scheduled. Out of the 8,430 children with a registered age of seven, 885 children were excluded for the following reasons: 638 for not having obtained parents' written consent, 146 because the results of the tests could not be read, 21 because their vaccination status was unclassifiable and 95 for miscellaneous reasons (not being at school on the survey days, refusing the test, delay in receiving permission, concurrent minor illness and history of preventive therapy). Fifteen out of the 95 children were nevertheless included because a previous tuberculin skin test reaction size diameter had been recorded. Hence, our analyses are based on 7,545 children (49.6% girls and 50.4% boys). Table 1 displays the distribution of children according to BCG vaccination status The ungrouped induration data is provided in an additional [see Additional file 1].

### II-Tuberculin and sensitin induration distributions

Using 5-mm interval categorisation (Table 2), it is clear that the proportion of children having large induration diameters is larger amongst BCG-vaccinated children. Regardless of the vaccination status, more people react to sensitin compared to tuberculin. Analyses of induration distribution by sex and local health area did not show statistically significant differences.

### III-Prevalence estimation using cut-off points

LTBI prevalences according to the commonly used cut-off points are displayed in Table 3. Tuberculin results show broad variation both according to vaccination status and the cut-off point method selected. However, sensitin results are quite stable regardless of vaccination status if strict criteria are used. If a less stringent cut-off point is chosen (including doubtful reactors, that is, those with 6–11 mm induration), this stability is lost (unvaccinated prevalence 11.59%, BCG-vaccinated prevalence 26.25%).

#### IV-Mixture models analysis

Among the theoretical distributions tested, the lognormal and Weibull distributions seemed too flexible to provide an adequate fit to our data, which are characterised by showing a high proportion of zero indurations and not having clearly identifiable modes for the components. In contrast, the normal mixture models provided a proper fit as reflected in comparatively good BIC results and good adjustment to the observed distribution. In Table 4 we provide the parameters' estimates of the component of interest for each mixture model.

#### Tuberculin

The LTBI estimated prevalence in the BCG-vaccinated group, although showing wide confidence intervals, is higher than expected, especially as compared with its counterpart prevalence figure in the unvaccinated group.

**Figure 1:**
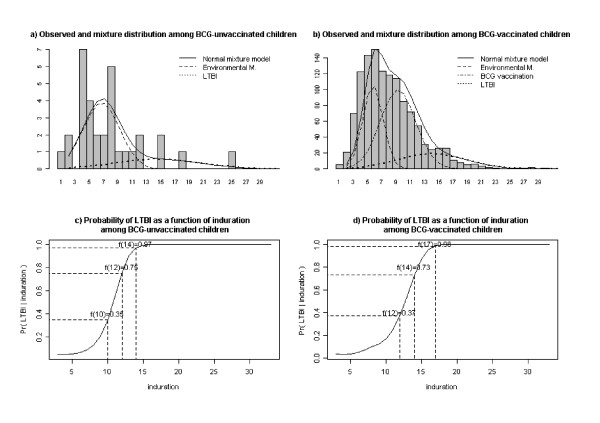
Induration distribution after tuberculin test and probability of LTBI as a function of induration diameter (mm) among unvaccinated children (on the left) and BCG-vaccinated children (on the right)

The observed induration distributions, the components of the mixture model and the normal mixture model are represented in figures 1a and 1b. On one hand, the fit of the mixture model is better in the BCG-vaccinated group, because it is based on many more people and three components are in use. On the other hand, in the unvaccinated group the components are more clearly distinguished. Finally, figures 1c and 1d show the probability of LTBI as a function of induration among unvaccinated and BCG-vaccinated children respectively. It is inferred from the first figure that indurations of 10 mm carry an estimated probability of infection of 35%, and it sharply arises as the induration increases from 10 to 14 mm, attaining a probability of infection of 97% for indurations of 14 mm (see Figure 1c). In contrast, the induration needed to consider LTBI with a probability of 98% among BCG-vaccinated people is 17 mm, 3 mm higher than among unvaccinated.

#### Sensitin

**Figure 2:**
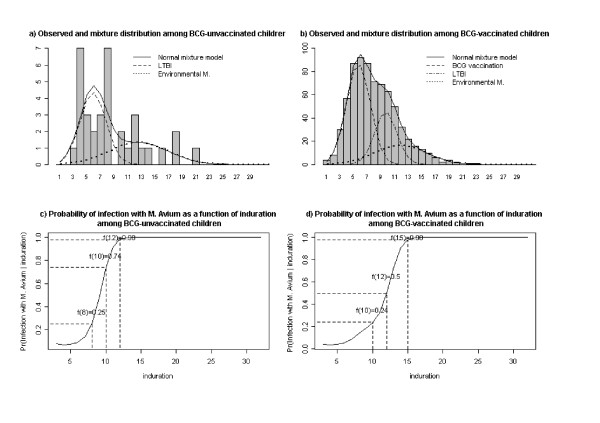
Induration distribution after sensitin test and probability of M avium infection as a function of induration diameter (mm) among unvaccinated children (on the left) and BCG-vaccinated children (on the right).

M avium estimated prevalence (Table 4) is very similar regardless of the vaccination status, around 7.5%. Similarly to figures 1a and 1b, figures 2a and 2b show a better fit of the mixture model in the BCG-vaccinated group. The relative influence of the last component seems to be bigger for the sensitin test than for the tuberculin test.

Finally, figures 2c and 2d show the estimated probabilities of M avium infection as a function of induration diameters among unvaccinated and BCG-vaccinated children respectively. The first figure shows that indurations of 8 mm carry a probability of infection of 25%, and it sharply arises as the induration increases from 8 to 12 mm, attaining a probability of infection of 98% for indurations of 12 mm. In contrast, the induration needed to consider M avium infection with a probability of 98% among BCG-vaccinated people is 15 mm, 3 mm higher than among BCG-unvaccinated people.

## Discussions and conclusions

We present the results of a tuberculin survey conducted in a western European population characterised by a low-intermediate TB incidence rate and high vaccination coverage. The prevalences of LTBI and M avium infection are estimated using different mixture models based on empirical-Bayes estimation methods and compared to traditional estimation approaches. Additionally, a decision-aid tool that capitalises on our own data and provides estimates of the LTBI probability as a function of induration instead of externally drawn cut-off proposals is presented. This approach and the results obtained shed some light on a complex issue with remarkable importance both from clinical and public health perspectives.

In a setting with low-intermediate tuberculosis incidence rates, high BCG coverage and a suspected presence of MNT, data of good quality is essential. For this reason, we designed this study taking special care in controlling the quality of all its procedures. Skilled personnel were enrolled, and a rigorous training process was undertaken in order to ensure homogeneous administration and reading of the derivatives [30]. Double and independent readings are not, as far as we know, a routine procedure in tuberculin and sensitin surveys.

Two features of this procedure deserve to be highlighted: the rapid execution of the fieldwork which ensures stability of conditions and the high participation rate (about 90%). Furthermore, the classification of vaccination statuses was very stringent, requiring at least two out of the three criteria to be positive: certificate of BCG vaccination, register confirmation and BCG scar [31].

Regarding the analysis of our TST results, it is remarkable how the method used influences the estimated LTBI prevalence (Table 3). Almost 7% out of the 5277 children presented an induration size greater than or equal to 10 mm., and therefore this cut-off point does not seem to be specific enough in the detection of LTBI due to the presence of NTM, BCG included [32]. When the "ø ≥ 14 mm × 1.22" method is utilised, almost 3% of the children are classified as infected by M tuberculosis. Using the mirror method [11,14,24], the LTBI prevalence would be 1.46%, which is more in agreement with tuberculosis rates in our region. Similar conclusions are reached by Salaniponi et al. [24] who also faced similar problems when applying these criteria in a different socio-demographic context.

Concerning environmental mycobacteria, there has not been any study so far in our country with sensitin using a sample as large as ours. Using the Palmer and Edwards criteria [13] referred to by Bosman [14], at least 5.2% out of the 2268 children who received sensitin as the first skin test would be infected with *M. avium*. These children were given TST one week later, and 3% of those who have had an induration size equal to or larger than 5 mm to the sensitin had null reaction to the tuberculin [33].

When comparing sensitin reactions with tuberculin ones using 5 mm-interval induration categories, the proportion of children with positive responses is clearly higher for the sensitin (Table 2). These findings provide additional support to the idea that exposure to environmental mycobacteria is common in areas with low tuberculosis infection rates and its effect should be accounted for when studying LTBI prevalence [34,35].

Because of the difficulty of disentangling the origin of the induration, we chose mixture models since they provide an appropriate framework in situations where a measurable variable (diameter of induration) is observed but the subpopulation (representing the main cause of skin test reaction) to which each individual belongs is unknown. From this analysis, we can obtain not only prevalence estimations but also information about the characteristics of each subpopulation and the individual classification probabilities. The estimation procedure of the parameters of a mixture model in general can be difficult when the number of components is large and/or when the component distributions overlap to a great extent. For this reason, it is more difficult to analyse BCG-vaccinated children than unvaccinated children, where convergence problems may arise in the estimation procedure. To minimise this problem, we analysed the data assuming several distributions for the components. Eventually, the normal mixture model provided both a reasonably good fit to the data and clinically interpretable estimates. We fitted the models using an empirical Bayesian analysis, which is a time-saving alternative to the full Bayesian approach used in this context by other authors [15], with the additional advantage that there is no need to include prior knowledge. To test this alternative estimation method for robustness, it has also been applied to data obtained from a rather different setting (summary data from the 1975 tuberculin survey in Korea analysed in [16] using full Bayesian techniques and kindly provided by HL Rieder) with very similar results and interpretation provided by both methods.

The interpretation of the mixture model results show that the prevalence of LTBI among unvaccinated children is about 1.5%. On the other hand, the prevalence of LTBI among BCG-vaccinated children is about 4.9%. These figures, although lower than those obtained with the 10 mm cut-off approach, still remain higher than we expected. We think that this fact could be due to the low expected prevalence in this region, which makes it difficult for the mixture model to estimate the relevant last component (attributed to LTBI) with sufficient precision. In spite of the fact that our study enrolled almost 90% of the target population, it may be possible that among non-participants LTBI would be more frequent, contributing to the imprecision of the last component. However, for populations with a higher presence of Mycobacterium tuberculosis, the model should perform better, even in BCG-vaccinated populations. This is reinforced by our findings regarding M. avium infection, with a higher prevalence in our study.

This mixture model approach, hence, in our view provides important information concerning the effect of NTM exposure on skin reactions and also about LTBI and M avium infection probabilities according to induration size (figures 1 and 2).

Focusing on the probability of LTBI (figures 1c and 1d), it seems that on average, exposure to previous BCG vaccine shifts around 3 mm towards greater induration size to attain the same probability of infection in our study population. In particular, to attain a LTBI probability of around 97%, the observed induration is 14 mm. for unvaccinated and 17 mm. for BCG-vaccinated children, results that were similar regardless of the distributional assumptions adopted. This finding agrees with current guidelines for LTBI treatment [2] and can be considered as a useful, induration size calibrated aid to be used in addition to other epidemiological and clinical information to make individual decisions.

Regarding *M avium *infection, some 7–8% of the children could be infected according to the mixture model results, without significant differences among BCG-vaccinated and unvaccinated, with both prevalences being higher than those obtained from the TST. BCG vaccine seems to have a similar effect on sensitin and tuberculin induration responses, requiring an upward shift of an average of about 3 mm to attain the same probability of infection.

Comparing the probability of infection with *M tuberculosis *and *M avium *as a function of induration, it is clear that the diameters needed to consider infection are lower for sensitin than for tuberculin. According to the results for the sensitin test, 8 mm of induration for unvaccinated and 10 mm for BCG-vaccinated children show a probability of *M avium *infection of about 25%, attaining a probability of infection above 98% with indurations of 12 and 15 mm for unvaccinated and BCG-vaccinated respectively.

In summary, we can conclude that the survey carried out using both tuberculin and sensitin skin tests supports the fact that the presence of Environmental Mycobacteria in our region cannot be neglected. The approach proposed here to analyse the results of the TST provides an alternative method for LTBI prevalence estimation based on the information obtained from our own local setting data instead of using external criteria and endorses current LTBI treatment decisions in populations similar to ours [2]. Recently developed and currently available blood tests represent the biggest change since introduction of the TST test for the diagnostic and therapeutic management of LTBI in developed countries [4]. CDC guidelines recommend utilising whole blood IFN-γ test QFT-G instead of TST although a combined use of both tests may increase the diagnostic yield insomuch as specificity is improved [36]. This combined strategy may also maximise cost-effectiveness [37,38], at least in adults as recent research in children suggests in them tests interpretation can be more complex [39]. If this is the case our method may add valuable information and we recommend its use to analyse TBC surveys, especially in areas with intermediate-high TBC prevalences in order to better deal with this still challenging problem.

## Competing interests

The author(s) declare that they have no competing interests.

## Authors' contributions

JIV had the original idea for this research and participated in its design and coordination, BI performed the statistical analysis and helped to draft the manuscript, VC participated in the design of the research and conducted the skin survey, JIP participated in the statistical analysis and helped to draft the manuscript, JT worked in the data magement process and helped to draft the manuscript and AU participated in the statistical analysis and helped to draft the manuscript. All authors read and approved the final manuscript.

## Pre-publication history

The pre-publication history for this paper can be accessed here:



## Supplementary Material

Additional File 1 Induration data set. Induration data set. Click here for file

Additional File 2 Codes for mixture models. Codes for the case of normal components for the freely available R software. Click here for file

## Figures and Tables

**Table 1:** Children's distribution according to BCG vaccination status. % are row percentages.

	**Unvaccinated**	**BCG-vaccinated**	**Total Analysed**
**Sensitin**	207 9.1%	2061 90.9%	2268 100%
**Tuberculin**	560 10.6%	4717 89.4%	5277 100%
**TOTAL**	767 10.2%	6778 89.8%	**7545** **100%**

**Table 2:** Distribution of tuberculin and sensitin induration according to vaccination status.

	**Unvaccinated**						**BCG-vaccinated**					
**Induration (mm)**	0	1–4	5–9	10–14	15–19	≥ 20	0	1–4	5–9	10–14	15–19	≥ 20
**Tuberculin**	527 94.1%	10 1.8%	15 2.7%	4 0.7%	3 0.5%	1 0.2%	3491 74%	218 4.6%	648 13.7%	266 5.6%	76 1.6%	18 0.4%
**Sensitin**	172 83.1%	8 3.9%	15 7.2%	8 3.9%	3 1.4%	1 0.5%	1334 64.7%	99 4.8%	406 19.7%	183 8.8%	35 1.7%	4 0.2%

**Table 3:** Different criteria for estimating prevalence of infection.

	**Tuberculin **(0.1 ml 2TU-RT23)			**Sensitin **(0.1 ml RS-10 M avium)
	{ø ≥ 10 mm} *	{ø ≥ 14 mm} × 1.22^†^	{ø = 17 mm}+2 × {ø ≥ 18 mm} (Mirror method)^‡^	{ø ≥ 12 mm}^§^
**Unvaccinated**	1.43%	0.87%	0.71%	4.35%
**BCG-vaccinated**	7.63%	3.05%	1.55%	5.29%
**Overall**	6.97%	2.82%	1.46%	5.20%

*Bosman et al. (1998) (14).

^†^Salaniponi (2004) (24).

^‡^Styblo (1992) (23) and Bosman et al (1998) (24).

^§^Palmer and Edwards (1958) (13).

**Table 4:** Estimation of the parameters of the mixture models for the tuberculin and sensitin test.

		**Component**	**Distribution**	**Mean**	**Variance**	**p (%)**	**95% CI (p)**
**Tuberculin Test**	Unvaccinated	LTBI	Normal	13.38	5.86	1.51%	(0.36, 3.10)
	BCG-vaccinated	LTBI	Normal	12.95	4.84	4.88%	(1.7, 5.8)
**Sensitin Test**	Unvaccinated	Environmental Mycobacteria	Normal	12.53	4.33	7.01%	(1.9,10.9)
	BCG-vaccinated	Environmental Mycobacteria	Normal	11.84	3.84	7.77%	(0.99,12.96)
