# Supplementary material for: Analysis of latent tuberculosis and mycobacterium avium infection data using mixture models
Source: BMC Public Health. 2006 Sep 28;6:240. doi: 10.1186/1471-2458-6-240 (PMC1599726; doi:10.1186/1471-2458-6-240)
Supplement: Additional File 2 — Codes for mixture models. Codes for the case of normal components for the freely available R software. [file 1471-2458-6-240-S2.doc]

**CODES FOR THE MIXTURE MODEL ASSUMING NORMAL COMPONENTS**

**#### Read the data set displayed using 2 columns; ###**

**#### 1^st^ column mm, 2^nd^ column frec (number of people with that induration) ###**

source("sv.dat") #dataset sensitin vaccinated

attach(sv)

datasv<-c(rep(mm, frec))

x.trab<-datasv

J<-length(x.trab); G<-3

**#### initial values for the parameters of the mixture (try with different recommended)**

p0<-length(x.trab[x.trab==0])/length(x.trab)

p<-c((1-p0)/3, (1-p0)/3, (1-p0)/3)

sig<-c(rep(sqrt(var(x.trab[length(x.trab[x.trab==0]):length(x.trab)])/3),G))

mu<-c(mean(x.trab[x.trab!=0][x.trab[x.trab!=0]<quantile(x.trab[x.trab!=0], 1/G)]),

mean(x.trab[x.trab!=0][x.trab[x.trab!=0]>quantile(x.trab[x.trab!=0], 1/G)& x.trab[x.trab!=0]<quantile(x.trab[x.trab!=0], 2/G)]),

mean(x.trab[x.trab!=0][x.trab[x.trab!=0]>quantile(x.trab[x.trab!=0], 2/G)]))

**#########################################**

conv.crit<-1; j<-1; g<-1; i<-0; G<-3; I<-4000

prob<-matrix(0,J,G+1)

old.params<-array(0,c(I,10))

**##########################################**

while(conv.crit>.00000001 && i<I ){

i<-i+1

old.params[i,]<-c(mu,sig,p,p0)

**############## E-step ####################**

for (g in 1:G){

prob[,g]<-p[g]*dnorm(x.trab[], mu[g],sig[g])/(p0*0^x.trab[]+p[1]*dnorm(x.trab[], mu[1],sig[1])+p[2]*dnorm(x.trab[], mu[2],sig[2]) +p[3]*dnorm(x.trab[], mu[3],sig[3]) )

}

prob[,G+1]<-1-sum(prob[,1:G])

**################# M-step #################**

for(g in 1:G){

mu[g]<- sum(prob[,g]*x.trab)/sum(prob[,g])

sig[g]<-sqrt(sum(prob[,g]*(x.trab-mu[g])^2)/sum(prob[,g]))

p[g]<-sum(prob[,g])/J

}

p0<-1-sum(p)

**########### log-likelihood #############**

logtest<-sum(log(p0*(0^x.trab[]) + p[1]*dnorm(x.trab[],mu[1],sig[1])+p[2]*dnorm(x.trab[],mu[2],sig[2])+ p[3]*dnorm(x.trab[],mu[3],sig[3]) ))

verosim[i]<-logtest

**############ convergence and results ###**

conv.crit<-max(abs(old.params[i,]-c(mu,sig,p,p0)))

}

print("n.iteraciones"); print(i); print("parameters"); print(round(c(mu,sig,p,p0,logtest),4))

**####### obtaining expected cases and probabilities for each induration size ######**

x<-c(0:32)

pi<-p0*0^x+p[1]*dnorm(x,mu[1],sig[1])+p[2]*dnorm(x,mu[2],sig[2])+p[3]*dnorm(x,mu[3], sig[3])

pi<-round(c(pi),6) ; pi1<-p0*0^x+p[1]*dnorm(x,mu[1],sig[1])

pi2<-p0*0^x+p[2]*dnorm(x,mu[2],sig[2]); pi3<-p0*0^x+p[3]*dnorm(x,mu[3],sig[3])

ni<-sv; fi<-ni$frec/length(x.trab)

ei<-pi*length(x.trab);

ei1<-pi1*length(x.trab); ei2<-pi2*length(x.trab); ei3<-pi3*length(x.trab)

**######## Figure plotting observed and mixture distribution ##############**

barplot(sv$frec[2:32], names=as.character(c(1:31)),space=0, cex.names=0.9, ylim=c(0,100))

lines(x[x>0]-0.5, ei[2:33], type="l", lwd=1.8,lty=1)

lines(x[x>0]-0.5, ei1[2:33], type="l", lwd=1.8,lty=2)

lines(x[x>0]-0.5, ei2[2:33], type="l", lwd=1.8,lty=4)

lines(x[x>0]-0.5, ei3[2:33], type="l", lwd=2.5, lty=3)

title("Observed and mixture distribution among BCG-vaccinated children")

legend(14,100,c("Normal mixture model" ,"BCG vaccination" ,”LTBI", “Environmental M."),lty=c(1,2,4,3),bty="n")

**######### Figure plotting probability of infection as a function of induration #######**

tubercu<-p[3]*dnorm(x, mu[3],sig[3])/(p0*0^(x)+p[1]*dnorm(x, mu[1],sig[1])+p[2]*dnorm(x, mu[2],sig[2])+p[3]*dnorm(x, mu[3],sig[3]))

plot(x[x>2], tubercu[x>2], type="l", ylab="Pr(Infection with M. Avium | induration) ", xlab="induration")

title("d) Probability of infection with M. Avium as a function of induration \n among BCG-vaccinated children")

#similar for other data sets, for unvaccinated (G=2) and other mixture models (changing dnorm by dweibul and so on)

.
